# Medical student perceptions of curricular influences on their wellbeing: a qualitative study

**DOI:** 10.1186/s12909-020-02203-4

**Published:** 2020-08-31

**Authors:** Christine Byrnes, Vaishnavi Anu Ganapathy, Melinda Lam, Lise Mogensen, Wendy Hu

**Affiliations:** 1 School of Medicine, Western Sydney University, Ainsworth Building, Goldsmith Avenue, Campbelltown, NSW 2056 Australia; 2 Westmead Hospital, Westmead, Hawkesbury Road, Westmead, NSW 2145 Australia; 3 Royal North Shore Hospital, Reserve Road, St Leonards, NSW 2065 Australia

**Keywords:** Medical students, Wellbeing, Mental health, Peers, Staff-student relationships, Curriculum, Assessment

## Abstract

**Background:**

Medical student mental health and wellbeing is highly topical and the subject of much research. While theoretically informed definitions of wellbeing abound, how do medical students themselves understand and perceive wellbeing? What aspects of the curriculum do they regard as affecting their wellbeing and mental health? This study explored these questions, and aimed to identify factors associated with student acceptability of wellbeing programs and interventions.

**Methods:**

All students at an Australian undergraduate medical school (*n* = 619) were invited to complete a qualitative online questionnaire between 2017 and 2018 following the introduction of several wellbeing initiatives, including “Wellbeing Days” (WBD). WBD allow students to take single absence days for self-care. Open-ended questions were asked about perceptions and experience of mental health and wellbeing, and views on interventions to improve wellbeing such as WBD. Thematic analysis was conducted across all responses. Three authors developed preliminary themes, which were then refined and confirmed by all researchers**.** Thematic saturation was achieved within data from the 68 respondents, which included participants from all cohorts.

**Results:**

Participants described wellbeing as positively experienced work/life balance, impacted by four factors; contact hours, peer relationships, staff relationships, and trust in how wellbeing initiatives were used. Long contact hours were deemed incompatible with self-care activities, maintaining employment, and seeking professional medical/psychological help. Peers could promote wellbeing by offering social and academic support, but also undermine wellbeing by being competitors. Degree of trust, engagement and communication with staff influenced acceptability of interventions. Participants viewed initiatives such as WBD favourably, but distrust of peers, and of staff, led to perceptions that WBD could be prone to misuse, or used for surveillance rather than support.

**Conclusion:**

Our findings suggest that wellbeing days which allow self-care, reduction in contact hours, and peer support may promote student wellbeing, but the acceptability of any interventions is influenced by relationships between staff and students, and in particular, trust in these relationships. We suggest strategies to strengthen trust and further research to investigate the relationship between trust and perceptions of wellbeing in self and peers.

## Background

Wellbeing and mental health are of great concern to Australian medical students [1] and medical students internationally**.** A national Australian survey of doctors and medical students [2] found medical students are at high risk for mental illness including depression (13.2% of males and 21.1% of females, ever diagnosed), anxiety (8.6% of males and 15.2% of females, currently diagnosed) and suicidal ideation (19.2% in the 12 months prior to the study). These findings are comparable to international prevalence rates [3].

Medical student wellbeing is often described within the context of factors that attenuate or increase the risk of mental illness [4], or in relation to prevalence of anxiety or depression [5]. Wellbeing is defined variably in the research literature. Definitions range from conceptual metaphors that incorporate multiple constructs, such as wellbeing as a ‘coping reservoir’ governed by personality, temperament, coping style, external stressors and social support [6], to measures using validated instruments of stress, anxiety, disinterest, motivation, and approaches to learning [7]. Given the Australian context of this study, we adopted a definition from a recent Australian and New Zealand medical educators consensus statement; wellbeing is “when individuals have the psychological, social and physical resources they need to meet a particular psychological, social and/or physical challenge [8].

Literature suggests that academic stress, less time with personal support networks, financial concerns, and exposure to human suffering and death are challenges which may precipitate medical student mental health decline [9]. Reluctance to prioritise mental health self-care is often compounded by stigma about mental illness amongst medical students, and fear of career consequences from seeking treatment [10].

In response to increased concern, recent initiatives include campaigns to reduce stigma, national events and many local programs [11, 12]. Graduates of Australia and New Zealand medical programs are expected to recognize their own health needs, and be aware of fatigue and stress management. Self-care is mandated in the curriculum [13].

Mental health absence days are a recent development at two Australian universities and in a high school setting in the United States [14–16]. Such days are based on the premise that during times of increased stress or poor mental health, students may benefit from a day free of attendance requirements. However, the relationship between medical curricula design and student wellbeing is not well-researched and little evaluation exists of interventions such as Mental Health Absence Days outside their original context [8]. This, together with varied definitions of wellbeing in different studies, suggests more foundational research is needed to inform research and evaluation of medical student wellbeing initiatives. Our study therefore aimed to examine how medical students understand and experience wellbeing and mental health, and the contextual influences which affect these perceptions, with a view to informing the design, evaluation and further research on curricular initiatives aimed at improving wellbeing.

Our research questions were:

1. How do medical students conceptualise wellbeing?
2. What do students identify as impacting on their wellbeing?
3. How do students perceive wellbeing interventions such as wellbeing absence days?

## Methods

### Study context

Our study was conducted in a five-year undergraduate Australian medical program located in an outer metropolitan medically underserved region in New South Wales with significant areas of socio-economic disadvantage. Students are preferentially recruited from the region, with the goal of broadening participation from students from non-traditional backgrounds. Students vary from those who directly enter after high school, to mature age students embarking on university study after an extended period of employment. Diversity of ethnic and parental occupational backgrounds reflect regional demographics and successive waves of migration. The region includes the largest Aboriginal urban community in Australia. Most (80%) students are Australian citizens, with the remainder being international. Central to the pedagogical design is experiential learning, supported by an attendance policy to ensure graduates meet accreditation standards for competence and patient safety, and reinforced by sanctions for non-attendance including reduced grades and risk of exclusion. In this medical program, the first two (‘preclinical’) years are largely on campus, with 1 day per week for hospital-based learning and another for self-directed study. Compulsory sessions include small group Problem Based Learning, professional development, hospital and laboratory based teaching. The final three (‘clinical’) years comprise full time clinical and community placements with students expected to be part of the service team for at least 35 h per week, with additional time required to complete online modules and self-directed study. Attendance requirements range from 34 weeks in first year, increasing to 43 weeks in final year, including examinations. One week of vacation is scheduled after examination periods, two or three times in each year.

In 2017, Wellbeing Days (WBD), co-designed with the medical student society, were introduced for students in the clinical years. These days were intended to assist students to monitor and manage their own mental wellbeing, with a secondary aim of identifying and assisting those students in need of additional support [14]. WBD were in addition to usual allowable absences for physical and mental illness and for special circumstances such as carer responsibilities. However, different to these, students were not required to submit independent verification, such as a certificate from a medical practitioner. WBD were not permitted on days when mandatory assessments were scheduled. Students were only required to submit a request indicating Wellbeing Day(s) as the reason, and there was no upper limit to the number taken. Students were informed that if an unusually high number were taken, the student would be invited to meet with the clinical program director to discuss whether additional support was needed. The intervention was considered by teaching staff and students as a distinctive change in how student wellbeing could be promoted, and awareness of the need for wellbeing increased [14].

### Study design

An online qualitative survey design was chosen, given the exploratory, sensitive and personal nature of the topic. Other than their year of enrolment, no other identifying details were requested. Survey questions were developed from the literature, piloted and administered using Qualtrics™. Participants were asked for their views on (1) wellbeing, (2) interventions for improving mental wellbeing, and (3) about wellbeing absence days (“WBD”) and other interventions [See Additional file 1]. No questions were mandatory (forced) and no word limit was applied.

### Participant recruitment

Students from all years in the medical program (*n* = 619) were invited to participate via promotional flyers, social media and email with a reminder at 3 months. Study information was provided and informed consent obtained prior to commencing, and submitting the survey. Participants were assured their participation was voluntary and confidential, and would not affect their relationship with the medical program. Ethics approval (H9989) was obtained through the Western Sydney University Human Research Ethics Committee.

### Data collection

Data were collected between October 2017 and February 2018. Of the 619 enrolled students, there were 150 questionnaire attempts of which 68 were completed and submitted. One participant elected to be interviewed face to face and their responses transcribed verbatim. Of the 68 completed surveys included in the data analysis, seven did not indicate their year of study.

### Data analysis

Data were analysed thematically guided by Braun and Clarke’s [17] approach. Three researchers (CB, ML, VG) read and categorised the responses in a process of familiarisation. CB and ML identified preliminary codes across the entire data sample, using computer assisted qualitative software QDA Miner Lite™. The codes and relevant text excerpts were then exported to MS Excel for further analysis using the constant comparative technique to compare responses to different questions, between different year cohorts and individuals. Repeated recurrence of themes was noted across the entire sample through iterative discussion and review. All researchers further refined and came to consensus on all the final themes. All researchers agreed that thematic saturation was achieved within the sample.

### Researcher positioning

Three researchers were students in the program at the time of the study (CB, ML, VG) and two were academic staff (LM, WH) with qualitative research expertise. These staff were not involved in the implementation and monitoring of WBD, or in data collection or analysis of any raw data for this study. All researchers were involved in student support and wellbeing policy development, either as part of a student society interest group, or as part of their program roles (school disability coordinator, associate dean).

The sensitive nature of the topic, and potential disclosures of mental illness and self-harm required careful consideration. The voluntary nature of the study was emphasized, and all participants were provided with information for medical and mental health counselling and telephone helpline support services. Participants were informed of the topics that would be covered prior to commencing the survey. The peer researchers conducting data collection and analysis received training on peer mental health support.

## Results

Participants were from all years of the program, predominantly from the first clinical clerkship, or third year, and the subspecialty fourth year of the program [see Table 1]. Participants described medical student wellbeing as a positively experienced balance of different life factors, including health (including elements of mental, physical, and emotional health), academic performance, personal circumstances, attitudes to self, and ability to cope with stress. Satisfaction with undertaking a career in medicine was considered part of their wellbeing. Four major themes emerged as impacting on wellbeing and acceptability of wellbeing initiatives: aspects of program design including *long contact hours*; *relationships and interactions with staff; relationships and interactions with peers*; and *trust and uptake of wellbeing initiatives* such as WBD. These are described below with illustrative quotes indicating the year cohort of the participant.

**Table 1:** Year cohort of participants

Student year cohort	Number of responses	Proportion of total sample (%)
Year 1	4	6
Year 2	7	10
Year 3	21	31
Year 4	20	30
Year 5	9	13
Not disclosed	7	10
TOTAL	68	100

. ***N*** **= 619**

### Long contact hours

Twenty participants, the majority from the clinical years, stated that contact hours affected their wellbeing. Concerns about full-time attendance requirement were strongly expressed; requirements were seen as overwhelming, and affecting their psychological wellbeing. Difficulty maintaining paid employment and resulting financial stress, incompatibility with activities of self-care were cited as factors. Terms such as “unsustainable”, “struggling”, “demanding” and “not dealing well” were used. Many described constraints on their personal time, with maintaining family and social relationships, physical health, pleasurable activities and hobbies, in addition to impact on paid work and home routines:

“I need a part time or less time option. I'm not dealing well with spending 8 hours in the hospital, coming home, going to work, trying to do chores and study around it*.*”

Participant 19, Third Year

“The long contact hours, and late shifts led to ‘medicine’ being seen as all-consuming, with insufficient time during the week and scheduled vacations to rest and recover:”

“…more holidays, and the reason is that hospital can be quite isolating and gruelling. Yes, we have weekends, but no-one can be naive enough to think that after long hospital hours from Monday to Friday on top of assignments, study and extracurricular commitments, that there is sufficient time to be adequately balanced*.*”

Participant 30, Third Year

“The quality and culture of the clinical environment could greatly influence how the attendance requirements were experienced. A setting where the supervising clinical team was seen to be unsupportive and mistreating students catalysed negative experiences, whereas well-planned and supervised placements leading to positive learning experiences were seen as time spent well, despite the long hours.”

“Factors affecting student wellbeing can include high stress levels, bullying and harassment by academic and clinical personnel, being time poor and unable to engage in self-care*.*”

Participant 61, Fifth year

“…time spent on placement facilitates our learning, interacting with other students, having clear direction with expectations and goals for placement”

Participant 15, Third Year

### Relationships and interactions with staff

Relationships with staff and perceived attitudes toward students from staff were cited as significantly impacting on wellbeing by participants in all years. Academic and wellbeing support was reported as being sought from clinical tutors, university staff, placement supervisors, administrative staff and wellbeing support services. Interactions with academic staff (cited by 12 participants), placement supervisors (10 participants), and administrative staff (11 participants) were described as key to accessing support, but there were divergent views on this experience.

Some participants reported increased anxiety following negative experiences of help-seeking, further affecting their wellbeing. Staff responses were described as “intimidating”, perceiving a “threat of punishment”, “disciplinary” tones, and that “they don’t take notice”, with some first year participants feeling unheard by staff. Some participants reported this apparent lack of interest, and poor communication as causing greater stress and distress:

“Admin staff at the university seem to almost enjoy keeping us in the dark… this is extremely stressful from an academic standpoint”

Participant 14, Third Year

“Negative perceptions were expressed about academic and administrative staff, with some participants only seeing academic staff as being responsible for disciplinary procedures, rather than to offer support. However, some preclinical students felt supported by administrative staff, and other clinical students highlighted positive interactions with clinicians:”

“Staff members have also been supportive and have provided opportunities to share”Participant 6, Second Year

“Many junior doctors or doctors on placements have taken me aside and told me I could approach them if there was something I saw that was overwhelming”

Participant 49, Fourth Year

### Relationships and interactions with peers

Twenty-seven participants reported relationships with their peers as influential on wellbeing, particularly in the first clinical year. Students described bonding with peers as a way to develop a sense of community within the student body, and thus a sense of safety and belonging while adjusting to being in a healthcare environment. The student community functioned as an informal peer support network, a place where student concerns could be heard through student advocacy:

“Because med school is so tough… we become very close to other med students”

Participant 19, Third Year

“I know medsoc [medical student society] runs events which promote wellbeing and socialising (which typically help your connection/belonging with other people).”

Participant 8, Second Year

“Conversely, a lack of peer bonding diminished participants’ sense of wellbeing. Clinical learning could be accompanied by social isolation when allocated alone to unfamiliar clinical departments:”

“…lack of closeness with fellow students due to the constant rotations, multiple hospital locations and lack of tutorials with students in the same group.”

Participant 58, Fifth Year

Further, competitiveness was sensed amongst peers, impacting negatively on wellbeing and worsening the sense of isolation:

“Spending 40hrs a week sometimes in a hospital surrounded by high achievers makes me feel inadequate and as though I need to work harder. This constant comparison to others, the high stress environment…”

Participant 21, Third Year

### Trust and uptake of wellbeing initiatives

All participants were aware of the recently introduced Wellbeing Days (WBD), more so than other supports and initiatives. More than half (*n* = 37) participants identified the WBD concept as “good in theory”, providing an option to “regenerate and recharge” that could reduce stigma about mental health and thus self-care.

“I think it is wonderful. Long before Wellbeing Days were officially recognised, students had been taking days off for mental health but being forced to come up with other supposedly more 'legitimate' sounding excuses. Often this meant feeling guilty for letting down our peers and faculty, and being made to feel lazy or just not cut-out for the work load.”

Participant 38, Fourth year

“I think it is incredibly symbolic and could really change peoples’ attitudes to what it means to succeed in medicine (i.e. not just working yourself to death at the expense of all other things in your life)”

Participant 59, Fifth year

However, many also highlighted barriers to their uptake, expressed fear of repercussions, a lack of awareness of how WBD were monitored, or “policed”. They related accounts of students being “called up” and of measures that were perceived as “punitive”. Some felt dissuaded from using WBD for these reasons, and expressed disappointment at the perceived mistrust from staff members:

"I know of a particular student in 3rd year who had 2 days off over the span…and was called in for a meeting because this was deemed as too many and caused concerns. [The student] was really angry about being questioned unnecessarily about their mental health and did not feel it was in a comforting, supporting way"

Participant 24, Third Year

The underlying concern was the attendance policy, which had become a focus of official staff-student interactions, such as regular reminders about compulsory sessions. It appeared that applying the policy affected wellbeing, implying that WBD and other wellbeing initiatives might not be needed if this was not the case:

“I think the attendance requirements in this medical degree are reasonable, but the policing of the attendance requirements is not. This is a degree to prepare us for a job that requires a great deal of responsibility. I do not understand why we need to be policed as though we are still school children if we have undertaken this responsibility”

Participant 61, Fifth Year

“Monitoring of attendance through WBD featured in anecdotal stories of students who had used WBD. Participants felt discouraged by such instances, feeling that they perpetuated mental illness stigma and signaled distrust of medical students:”

“I know that people who have been followed up have felt like tabs are being kept on them to a) make sure they aren't just not turning up and using it as an excuse and b) to mark them as having ‘mental health issues’”

Participant 31, Third Year

“Some participants expressed a general lack of trust of students in faculty members and as an extension, wellbeing initiatives endorsed by the school.”

“…to take a day that is supposed to be, and I quote, '-a personal day off with no questions asked' and then to renege without warning and start punitive action against students taking ‘too many’ wellbeing days….completely defeats the purpose of the policy and in fact only further severs the trust and amenability between students and the administration”

Participant 26, Third Year

Misuse by other students was also a factor which could erode staff trusting students to utilise WBD responsibly. Participants felt frustrated if they perceived that the consequences for unprofessional behaviour were inadequate. Using WBD for spurious reasons by peers for non-attendance was reported, with some perceiving that their misuse could lead to individual gain.

“A sense of injustice - when some students are very lax with attendance but then get better marks than someone who has been present and involved in the rotation”

Participant 22, Third Year

However, others expressed greater trust in school processes and staff intentions, accepting that attendance monitoring was necessary to avoid abuse of WBDs, and that mutual trust between students and staff was necessary for initiatives to work well:

“It requires honesty from students (nil abuse of 'days off') and understanding and compassion from the Medical School”

Participant 33, Fourth year

## Discussion

Our study was conducted within the context of introducing a distinctive initiative to promote student wellbeing, providing an opportunity to gather student views on wellbeing and the influences on their wellbeing whilst studying medicine. Participants described wellbeing as a multifaceted construct, encompassing a balance of experiences and beliefs including their personal health, academic performance, life circumstances and stress management skills and self-efficacy. This is consistent with wellbeing as an expansive concept and state of being [6, 18] similar to the World Health Organization concept of quality of life [19]. Findings further identified program design influences on student wellbeing; key to these was the quality of relationships between students and staff and between students and peers, thus impacting on attitudes towards formal wellbeing initiatives and their subsequent uptake. Previous research has noted that medical student and staff perceptions and stigma about mental illnesses can affect the success of student support initiatives [20]. While our findings represent student perceptions at one point in time, such perceptions may fluctuate over time and progression through key training transitions [21], rotation of staff and students, curricular changes, and prevailing community attitudes to medical student and doctor mental health. In our study, students experiencing their first clinical year, strongly expressed the impact of the new learning experiences on their wellbeing. While such experiences are well documented [22], we found that these effects persisted throughout the study year and into the final years of the course.

We explore below the program features which participants described as influencing their wellbeing and their views towards wellbeing initiatives.

### Contact hours and attendance

Full-time attendance requirement was repeatedly identified as impacting negatively on wellbeing. Although this could be moderated by trust and communication between staff and students and the quality of the learning environment, more often students cited the quantum of hours required. Participants reported limits on time spent with family or friends, diet, exercise, access to medical care and paid employment. Low levels of physical activity during medical school are associated with higher emotional exhaustion and burnout [23], and exercise programs for trainee doctors can reduce burnout [24]. Likewise, paid employment and maintaining an income can reduce financial stress while providing an outlet, also decreasing the likelihood of burnout [25]. In our study, the stress of increased hours when transitioning to clinical learning did not wane, with students into the final years reporting similar impacts. Our findings suggest that clearer guidance and achievable expectations for each placement may prevent significant student stress and lead to improved academic performance and wellbeing. Furthermore, supportive clinical environments and re-design of processes such as monitoring of attendance requirements may reduce negative impacts on wellbeing.

The relationship between attendance hours and medical student wellbeing is rarely researched. Slavin et al. demonstrated lower rates of depression and anxiety in medical students after overall contact hour reduction, albeit as one component of a larger curricular overhaul [26]. One Australian medical school implemented a reduction in contact hours by 20% into their curriculum, resulting in no significant changes to summative assessment scores or year completion rate over 3 year cohorts [27]. One systematic review showed that students required to stay for overnight call were more likely to experience burnout, and this was attributed to the long hours [28]. Restricting duty hours reduced levels of emotional exhaustion and burnout in a systematic review of surgical residents [29]. Our study suggests a reduction, or greater flexibility of contact hours could improve wellbeing. Once established, wellbeing absence days could provide this flexibility. However, participation and engagement is also necessary for learning and has been found to relate to academic performance [30]. The reduction of resident work hours has led to debates about quality of learning [31–35]. The absolute necessity for graduates to reach a minimal level of competence for safe supervised practice means that reducing contact hours requires an evidence based and measured approach with frequent re-assessment of student wellbeing together with learning outcomes and performance.

### Relationships with peers

Peer relationships appeared influential. Respondents named positive effects from the social support of belonging to a strong student community and sharing experiences; and negative effects from perceived competition, isolation and concerns about misuse of support initiatives. Support from peers is associated with a higher degree of resilience [25] and students often prefer the support of their peers over institutional services [36]. Being part of a social group and identifying as a medical student within that group is associated with improved wellbeing [37]. Group cohesion encourages collaboration, with members working synergistically to improve wellbeing [37].

Third year students in their first clerkship year particularly emphasised the significance of peer relationships for wellbeing. During clinical years, students are placed singly or in pairs to clinical teams, and rotate to different teams every 4–5 weeks. For third year students, this experience was in stark contrast to the stable membership of small, tutorial groups during the first 2 years. Being separated from usual peer social groups is known to cause stress [38]. Isolation on clinical placements, exacerbated by a sense of alienation from being in an unfamiliar environment and suboptimal supervision from clinical teams, could be reduced through peer and near-peer support and sharing of experiences. Peer support could be particularly important during placement transition periods, which are known to trigger stress, worsened student perceptions of the learning environment and work-life balance [39]. Our findings support the introduction of near-peer support from junior doctors. Greater efforts may also be required to address the isolation and disengagement some students experience during clinical placements [40]. Nevertheless, the evidence is limited and mixed for programs aimed at enhancing and providing access to peer support [41].

Participants also reported experiencing negative impacts from competition between peers, through stimulating poor self-appraisal in comparison to others, and feelings of inadequacy. Reorienting assessments so there is less focus on grading and rankings, with rewards for collaborative learning [38] can lead to social and psychological benefits [40].

### Staff attitudes towards students

Our findings suggest that mutually trusting relationships between staff and students could promote wellbeing per se, as well as enhance engagement and uptake of wellbeing initiatives.

Participants reported wide variation in relationships with staff. Participants who reported negative experiences expressed concerns about the perceived disciplinary and punitive nature of their interactions, attributing these to their poor wellbeing. The opposite notion also held; those who related positive experiences described them as supportive of wellbeing. Previous research has shown that student perception of support and interest in students’ education by faculty is associated with increased odds for recovery from burnout [25], and clinical supervisors can serve as sources of stability for medical students [42].

As staff are information gatekeepers to many services, student mistrust of staff motives may dissuade students from approaching staff and thus participating in learning and support programs [43]. Engagement has behavioural, emotional and cognitive elements; students may be positively or negatively engaged along different parameters, or disconnected entirely [44]. Better understanding of the relationship between trust and student engagement is needed to build appropriate interventions and programs [45]. Effective training for staff and students about mental health, promotion of student support services, and to identify and address student distress are needed in Australian medical schools [45]. Training may lead to greater awareness of how well-meaning staff responses may be perceived by students as mistrust, thus affecting subsequent decisions to seek help. As participants noted, an environment of mutual trust between students and staff empowers students to develop into effective learners [46] and could foster wellbeing.

### Trust and student wellbeing initiatives

Our study suggests that initiatives such as WBD, aimed at promoting student choice and self-management, require a supportive, trusting environment to achieve their aims. While some participants noted that WBD could reduce mental health stigma, participants predominantly raised concerns about taking WBD. Trust appeared to influence their responses; some felt that staff did not trust students to use WBD responsibly, resulting in a perception of being ‘policed’ and exacerbating their mistrust of mental health school initiatives. For others, the possibility of being approached by staff after taking WBD and fear of consequences from disclosing poor mental health discouraged WBD uptake. Previous poor experiences, stigma around mental health and career impacts are known to affect help seeking [2, 36]. Unsurprisingly students are less likely to be open about their mental health to support staff who are also involved in their academic or professional evaluation [36, 42, 47]. Where possible, a separation of such roles is required for best practice. Dedicated support staff, separate from the assessment and course delivery is likely to be more effective, with psychiatrists attached to medical schools being one option [48]. In situations where a designated support position is limited or unavailable, general staff training and upskilling is suggested. In medical schools, student support is often an informal role; consequently, staff may base their support on past experiences and their perceptions about individual students [49]. The emerging research on staff roles could be incorporated into training that provides opportunities for reflective practice, adapted for sites, curriculum requirements and service availability [50]**.**

In addition to training that incorporates reflection on how policies and staff actions may impact on trust,

inclusion of student perspectives in all aspects of program design, delivery and evaluation is likely to build trust. In the study setting, accreditation standards require schools to describe how students are consulted and included in program delivery and management [13]. Crucial to maintaining trusting relationships is follow-up with students and investment in student inclusion over time [51], underpinned by regular communication with the student body that closes the loop on any student recommendations. This may improve student confidence in institutional policies and processes and motivate more students to engage with program development and initiatives such as WBD.

### Curricular implications

Policies to address the stress and sense of distrust associated with rigid attendance requirements, and to increase flexibility in attendance and assessment, could promote student self-care and offer medical schools more scope to adapt support for students with unique challenges [52]. Curricula that allow time and space to for students to reflect and foster a sense of purpose and meaning from their studies can reduce depression and anxiety [53].

WBD were intended to assist students to develop self-care and time management skills, which are common to professionalism graduate learning outcomes worldwide [5, 13, 54]. However, an unintended consequence may be expectations of flexible resident work hours which are not feasible in most clinical workplaces. To support the intended use and uptake of strategies such as WBD, learning activities in time management and its connection to professionalism as new graduate doctors could be included in professional development curricula. For workplace relevance, learning plans and workplace-based assessments conducted by clinical placement supervisors could provide learning opportunities that support the transition to graduate practice [54].

### Limitations

Our findings are context-specific so readers should interpret them against our description of the study context when considering transferability to their own student cohorts and institutions. Nevertheless, participants reported similar impacts, such as the stress of transitioning to clinical learning, as reported elsewhere. The relationship between *student* wellbeing, faculty and peer relationships and trust has not been reported, although associations between staff wellbeing and organisational culture are well known. This suggests that further research could confirm and explore the mechanisms for how medical school and clinical service cultures affect student wellbeing, and develop a model that could be applied in other settings.

The majority of participants were from the first clinical clerkship, or third year of the program, and the subspecialty fourth year, which students commonly find more challenging due to longer attendance hours and volume of new content. However, similar impacts were reported by participants in pre-clinical and final years of the program. Participants were likely to include those with greater interest, and more experience of mental health concerns in the medical school. This may have increased the reports of negative impact from full time attendance. As the study gathered data in one time period, it is not known whether and how many individuals will adjust to the attendance requirements over time. It may be that only those with negative effects participated. While the sample was a minority of the entire student body, thematic saturation was established with less than the 68 responses, although all completed submissions were analysed to identify possible outliers.

In this anonymous survey, student status was not able to be verified. It is possible that some participants were not authentic students. However, this potential exists with most online surveys and participants were barred from logging in more than once with the same internet protocol (IP) address. Our analysis of all completed responses showed a commonality, with no unusual outliers. The survey was open only between October 2017 and February 2018, reducing the opportunity of widespread public dissemination. One drawback of anonymity is being unable to further support those individuals who expressed marked negativity, a possible sign of depression and anxiety, other than general information about support services.

The non-mandatory open-ended questions were intended to provide a safe forum for participants to provide sensitive information, but meant that probing responses for depth and triangulation of reported events were not possible. Iterative discussion and interrogation of themes with all members of the research team, returning to the data repeatedly to ground any findings and interpretations, strengthened the credibility of the study findings. Being led by peers, students may have felt more comfortable participating and providing open and frank responses. The student researchers were however, known by the student body to be part of the team which co-designed the WBD initiative.

## Conclusion

Our study presents a snapshot of how medical students conceptualise and experience wellbeing, in the context of introducing a distinctive initiative aimed at promoting mental well-being self-management. Participants described wellbeing as a multifactorial state of being, affected by program features such as long and inflexible contact hours and unclear learning guidance, perception of supportive or negative staff-student relationships and peer relationships. Initiatives such as wellbeing days may address the negative effects of high contact hours, but their effectiveness can be influenced by trust between students, peers and staff. Our findings suggest re-design of attendance requirements, enhancing the quality of learning environments, peer and near-peer support mechanisms, curricular alignment and modifying assessment may improve student wellbeing. Staff training, inclusion of student voices in program development and evaluation feedback could promote trusting relationships.

Future research could focus on the relationship between contact hours, organisational culture, wellbeing and performance in medical students over time. Developing a theoretical model to explain the qualities of contact hours that optimise wellbeing and learning could assist in transferability of findings to other contexts, and address the limitations of wellbeing research that is short term and based on single institution program evaluations [20]. Although sustained programs are challenging to implement, research on the ongoing effects of support programs, using theoretically informed study designs to identify contextual features that impact on wellbeing, is needed.

## Supplementary information

**Additional file 1.** Appendix 1: Participant Questionnaire.

## Data Availability

The raw data collected for this study are not publicly available due to the personal nature of the topic and potential for participant re-identification. Aggregate de-identified data is available on reasonable request.

## Abbreviations

WBD: Wellbeing day
