# Supplementary material for: Medical student perceptions of curricular influences on their wellbeing: a qualitative study
Source: BMC Med Educ. 2020 Aug 31;20:288. doi: 10.1186/s12909-020-02203-4 (PMC7457773; doi:10.1186/s12909-020-02203-4)
Supplement: Supplementary file 1 — Additional file 1. Appendix 1: Participant Questionnaire. [file 12909_2020_2203_MOESM1_ESM.docx]

**Appendix**

Appendix 1: Participant Questionnaire

What does ‘medical student wellbeing’ mean to you? Can you give examples?

What factors do you think contribute to or affect medical student wellbeing?

In your experience, how have medical schools, health services and clinical placement settings improved student and trainee mental health and wellbeing?

What do you think about the idea and impact of medical students taking Wellbeing Days? (If you are a student and have taken a wellbeing day, what was your experience of it?)

Have you heard any positive or negative feedback from others about the use of Wellbeing Days? (Can you name the group (without identifying individuals)? Why do you think they felt this way?)

The following information is for demographic purposes only, and will not be used for identification: Are you a Student/Professional staff member/Academic staff member

What other strategies do you think should be considered to improve medical student wellbeing?

What factors contribute to or affect your own wellbeing?
